# Risk-stratification in febrile infants 29 to 60 days old: a cost-effectiveness analysis

**DOI:** 10.1186/s12887-021-03057-5

**Published:** 2022-02-03

**Authors:** Kathleen A. Noorbakhsh, Sriram Ramgopal, Nancy S. Rixe, Jennifer Dunnick, Kenneth J. Smith

**Affiliations:** 1 Department of Pediatrics, University of Pittsburgh Medical Center, Children’s Hospital of Pittsburgh, 4401 Penn Ave, Pittsburgh, PA 15224 60611 USA; 2 Division of Emergency Medicine, Ann & Robert H. Lurie Children’s Hospital of Chicago, Northwestern University Feinberg School of Medicine, 225 E. Chicago Ave, Chicago, IL 606111 USA; 3 Department of Medicine, University of Pittsburgh Medical Center, 200 Meyran Ave, Pittsburgh, PA 15213 USA

**Keywords:** Infant fever, Economic analysis, Clinical prediction rules, Serious bacterial infection, Neonatal sepsis

## Abstract

**Background:**

Multiple clinical prediction rules have been published to risk-stratify febrile infants ≤60 days of age for serious bacterial infections (SBI), which is present in 8-13% of infants. We evaluate the cost-effectiveness of strategies to identify infants with SBI in the emergency department.

**Methods:**

We developed a Markov decision model to estimate outcomes in well-appearing, febrile term infants, using the following strategies: Boston, Rochester, Philadelphia, Modified Philadelphia, Pediatric Emergency Care Applied Research Network (PECARN), Step-by-Step, Aronson, and clinical suspicion. Infants were categorized as low risk or not low risk using each strategy. Simulated cohorts were followed for 1 year from a healthcare perspective. Our primary model focused on bacteremia, with secondary models for urinary tract infection and bacterial meningitis. One-way, structural, and probabilistic sensitivity analyses were performed. The main outcomes were SBI correctly diagnosed and incremental cost per quality-adjusted life-year (QALY) gained.

**Results:**

In the bacteremia model, the PECARN strategy was the least expensive strategy ($3671, 0.779 QALYs). The Boston strategy was the most cost-effective strategy and cost $9799/QALY gained. All other strategies were less effective and more costly. Despite low initial costs, clinical suspicion was among the most expensive and least effective strategies. Results were sensitive to the specificity of selected strategies. In probabilistic sensitivity analyses, the Boston strategy was most likely to be favored at a willingness-to-pay threshold of $100,000/QALY. In the urinary tract infection model, PECARN was preferred compared to other strategies and the Boston strategy was preferred in the bacterial meningitis model.

**Conclusions:**

The Boston clinical prediction rule offers an economically reasonable strategy compared to alternatives for identification of SBI.

## Background

Fever is one of the most common chief complaints in the emergency department (ED), comprising 10-20% of all pediatric ED visits [1–3]. Among infants ≤60 days old, the prevalence of serious bacterial infections (SBI), including bacterial meningitis, bacteremia and urinary tract infection (UTI), in the setting of fever ranges from 8 to 13% [4–9]. Such infections pose potential risk of morbidity and mortality if not diagnosed [4–9]. The evaluation and management of young febrile infants involves extensive diagnostic testing, frequently followed by hospitalization and antibiotic therapy [10].

.Balancing the costs of medical evaluation and treatment, particularly unnecessary hospitalization, with the risks of misdiagnosis poses a clinical challenge. Over the last 40 years, multiple clinical prediction rules have been published to identify a cohort of infants at low risk of SBI [11–17]. Older prediction rules require routine lumbar puncture and cerebrospinal fluid (CSF) testing [15–17]. More recent prediction rules do not require CSF testing for risk stratification and offer improved diagnostic accuracy [15–18]. These rules carry the potential to improve clinical outcomes, decrease variation in care, and reduce high costs associated with the evaluation and management of febrile infants [4, 7]. The cost-effectiveness of published clinical prediction rules in this population is unknown. The decision to adopt a clinical prediction rule to evaluate febrile infants must be weighed against the cost and effectiveness of established risk-stratification strategies.

In this investigation, we use decision modeling techniques to evaluate the cost-effectiveness of different strategies to identify infants with SBI in the ED.

## Methods

### Study design

We created a decision-analytic Markov model to simulate a hypothetical cohort of infants 29-60 days old presenting to an ED using the following eight published risk-stratification strategies: Boston, Rochester, Philadelphia, Modified Philadelphia, Pediatric Emergency Care Applied Research Network (PECARN), Step-by-Step, Aronson, and clinical suspicion (Table 1) [11–18]. Selection of strategies was limited to those developed to identify infants at low risk of SBI and with published sensitivity and specificity data. All analyses used secondary data from the medical literature or from online US databases without identifiable patient information. The decision model was programmed in TreeAge Pro 2016 (TreeAge Software, Inc., Williamstown, MA).

**Table 1:** Strategy sensitivity and specificity for serious bacterial infection, required testing, and low-risk criteria

Strategy name	Parameters, Point estimate, % (Range)						Diagnostic testing					Low-risk criteria
	Urinary tract infection		Bacteremia		Bacterial meningitis							
	Sensitivity	Specificity	Sensitivity	Specificity	Sensitivity	Specificity	UA	CBC	CSF	CRP	PCT	
Rochester [13, 15, 19, 20]	91 (76-98)	46 (29-56)	90 (71-99.9)	48 (23-71)	99.9 (95-99.9)	39 (27-51)	●	●				Urine WBC < 10/hpf Blood WBC 5000-15,000/mm^3^ Band neutrophils< 1500/mm^3^
Philadelphia [17, 19, 21]	99.9 (97-99.9)	32 (21-43)	96 (86-99.9)	27 (10-43)	99.9 (95-99.9)	32 (20-42)	●	●	●			Urine WBC < 10/hpf Blood WBC < 15,000/mm^3^ Band:neutrophil ratio < 0.2 CSF gram stain negative
Boston [16, 22]	90 (84-94)	56 (54-58)	79 (59-92)	53 (51-55)	99.9 (99-100)	53 (50-55)	●	●	●			Urine WBC < 10/hpf Blood WBC < 20,000/mm^3^ CSF WBC < 10/hpf
Modified Philadelphia [13]	–	–	92 (83-96)	35 (26-41)	99.9 (80-99.9)	23 (19-28)	●	●				Urine WBC ≤5/hpf Blood WBC 5000-15,000/mm^3^ Band:neutrophil ratio < 0.2
Step-by-Step [11]	99 (97-99.9)	56 (53-59)	91 (82-96)	47 (45-49)	99.9 (95-99.9)	46 (43-47)	●	●		●	●	Urine WBC = 0 PCT < 0.5 ng/mL, ANC < 10,000/uL, CRP < 20 mg/L
PECARN [12, 23]	95 (87-99.9)	58 (52-63)	92 (73-99.9)	52 (44-59)	86 (29-99.9)	51 (43-55)	●	●			●	Urine WBC = 0 PCT < 1.71 ng/mL ANC < 4090/uL
Aronson [14]	–	–	98 (91-99.7)	36 (30-42)	98 (92-99.9)	34 (28-40)	●	●				No fever in ED UA < 5 WBC/hpf ANC < 5185/uL
Clinical suspicion [18]	78 (63-93)	65 (50-80)	83 (28-91)	37 (30-83)	93 (86-99.9)	35 (20-50)						Risk of SBI < 1%

*UA* urinalysis, *CBC* complete blood count, *CSF* cerebrospinal fluid, *CRP* c-reactive protein, *PCT* procalcitonin, *WBC* white blood cell count, *SBI* serious bacterial infection, *ANC* absolute neutrophil count, *PECARN* Pediatric Emergency Care Applied Research Network, *ED* emergency department

Our base case was a 40-day old term infant presenting to the ED with fever. A “base case” serves as the most likely scenario and lays the groundwork for model assumptions. All infants were assumed to be “well-appearing” and without localizing signs of infection. By selecting these characteristics, our base case infant met evaluation criteria and low-risk history criteria for each risk-stratification strategy. Infants < 29 days of age were excluded as most clinical prediction rules consider this age group to be inherently not low risk.

A Markov model consists of mutually exclusive “health states.” Simulated individuals reside in one health state at a time and can transition between health states as designated in the model. For this study, we considered five health states: 1) well, 2) SBI with medical treatment, 3) misdiagnosed SBI with no medical treatment, 4) misdiagnosed well infant with medical treatment, and 5) death. Infants in the simulated population were initially categorized as low risk or not low risk using each strategy. Infants with SBI who were misidentified as low risk had an increased risk of death above baseline [29–32]. Those remaining alive underwent reevaluation and medical treatment. Infants who underwent medical treatment were assumed to make a full recovery. Infants without SBI who were miscategorized as not low risk were assumed to undergo hospitalization without complication. Low-risk criteria were defined according to each clinical prediction rule (Table 1). For clinical suspicion, low-risk was defined as a risk of SBI < 1% as determined by the treating physician and was assumed to be assigned after history and physical exam but before obtaining diagnostic testing [18]. The threshold of 1% was selected based on a previous study [18]. For the Aronson strategy, we used a score < 2 to define low risk [14]. For the Boston strategy, empiric ceftriaxone administration for all low-risk infants was assumed to be protective against worsening infection and death [16].

**Fig. 1:**
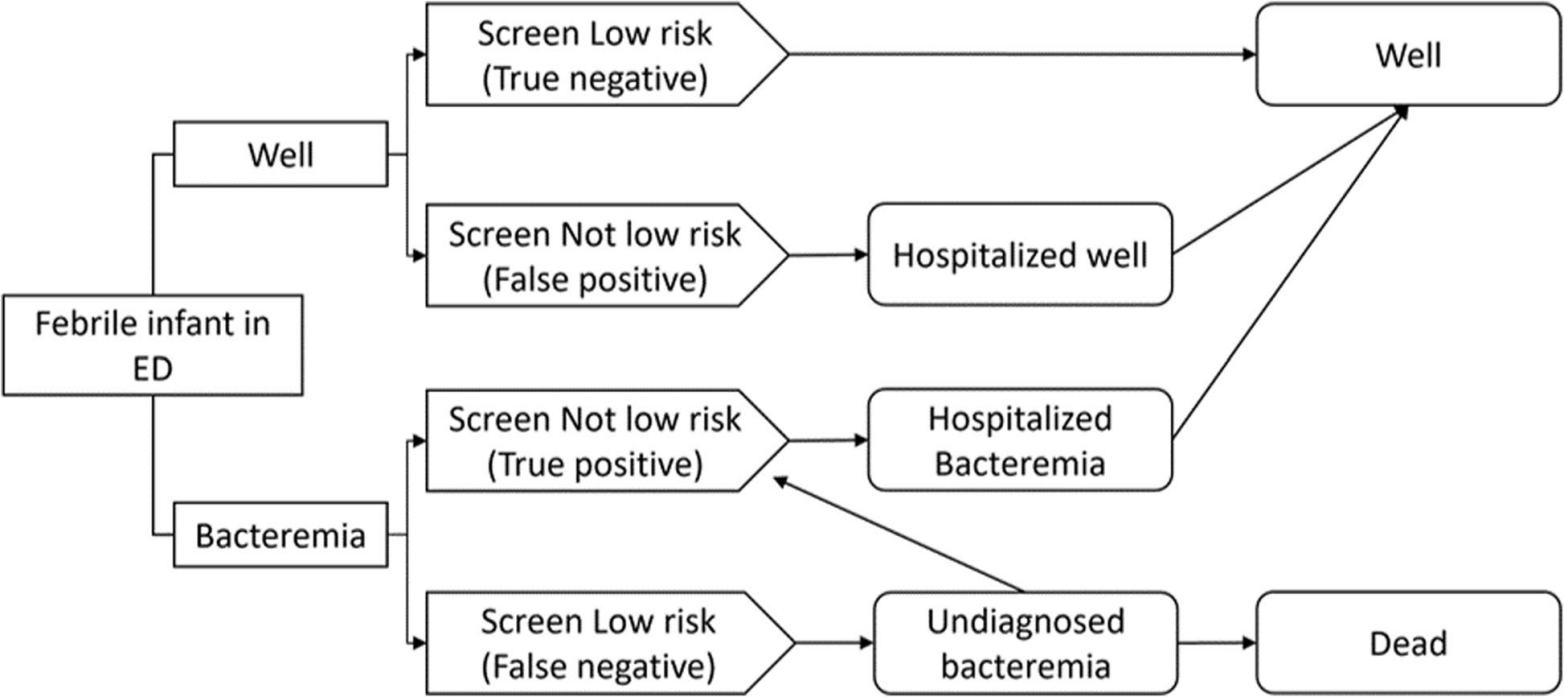
Model schematic for bacteremia. Infants begin the model in the emergency department with a fever, either with or without bacteremia and undergo risk-stratification. Infants with bacteremia who are misidentified as low risk have an increased risk of death. ED, emergency department

Due to significant variations in the reported prevalence [4–9], complications [8, 9, 30–36], and costs of treatment [37] for UTI, bacteremia, and bacterial meningitis, our primary model focused on bacteremia, with secondary models for UTI and bacterial meningitis. We chose bacteremia for our primary model, as it is more prevalent than bacterial meningitis and carries higher risks of morbidity and mortality than UTI [4–9, 30–36]. In the bacterial meningitis model, misdiagnosed infants with bacterial meningitis had an increased risk of death compared to the bacteremia model [9, 30–32]. In the UTI model, misdiagnosed infants with UTI returned to the ED and were treated, with a small proportion developing bacteremia [33–35]. There was no increased risk of death in the UTI model [33]. The Aronson and Modified Philadelphia prediction rules do not report sensitivity or specificity for UTI and were not included in the UTI model. We constructed a decision tree for each model of interest. A simplified version of the bacteremia decision tree is presented in Fig. 1.

### Model input variables

Input parameters for probabilities, costs, and outcomes are presented in Tables 1 and 2. For each variable, we included an estimated 95% probability range. We conducted a review of published literature to identify rates of outcomes for febrile infants and to identify measures of diagnostic accuracy for prediction rules (Table 1). Probabilities of outcomes from misdiagnosed bacteremia were derived from previously published literature, with ranges that accounted for variation among sources and uncertainty given paucity of data in the post antibiotic and vaccine eras [31]. All-cause mortality was estimated using U.S. National Center for Health Statistics life tables [38].

**Table 2:** Bacteremia model inputs: Baseline parameter values and ranges

Variable	Point estimate	(Range)
*Probabilities*		
Risk of bacteremia [7, 11–13, 31]	1.7%	(0.01-3.7%)
Risk of death, delayed antibiotics in bacteremia [29–31]	10%	(0-25%)
*Costs*^*a*^		
ED visit [39]	$553	(442-664)
Hospitalization [37]		
Infectious condition ruled out	$5550	(4440-6660)
Urinary tract infection	$5382	(4305-6458)
Bacteremia	$26,031	(20,825-31,237)
Meningitis	$29,464	(23,571-35,357)
Blood culture [27]	$12	(10-14)
CBC with differential [27]	$12	(10-14)
C-reactive protein [27]	$18	(14-22)
Procalcitonin [27]	$36	(29-43)
Urinalysis [27]	$4	(3-5)
Urine culture [27]	$11	(9-13)
Lumbar puncture [27]	$81	(65-97)
CSF culture [27]	$12	(10-14)
CSF gram stain [27]	$6	(5-7)
CSF testing, other [27]	$83	(66-100)
*Utilities*		
Well newborn [39]	0.95	
Hospitalization [39]	0.88	(0.58-1.0)
Bacteremia [24]	0.71	(0.4-1.0)
Lumbar puncture [25]	^−^ 0.1	(0.0 - ^−^ 0.5)
Disutility of death [38]	30.98	

^a^Costs are in 2016 U.S. dollars

*ED* emergency department, *CSF* cerebrospinal fluid, *CBC* complete blood count

Sensitivity and specificity of each clinical prediction rule for bacteremia, UTI, and bacterial meningitis, were hand calculated by two of the authors (KAN, SR) based on published data [11–23]. When more than one study for a prediction rule was identified, the mean values for calculated sensitivity and specificity were used. Range was based on 95% confidence interval for prediction rules with a single data source. For prediction rules with externally validated data, the range was broadened to include values from all calculated 95% confidence intervals.

Costs included direct medical costs of ED visits, diagnostic testing, and medical treatment (Table 2). Costs of hospitalization were based on the mean value for each diagnosis for children less than a year of age, as reported by the Healthcare Cost and Utilization Project [37]. All costs were adjusted to 2016 US dollars based on the medical cost component of the Consumer Price Index [26]. We selected 2016 as this was the most recent year available for US inpatient healthcare data from the Healthcare Cost and Utilization Project [37].

Health state utilities were assigned a value of 0-1, with 0 equivalent to death and 1 representing perfect health [28]. Utility values associated with various outcomes were drawn from the literature [24, 25, 39, 40]. When quality-of-life studies were not available for this age group, we used quality-of-life estimates from older populations. Infant mortality was factored as a lifetime disutility, meaning that the lifetime loss of quality-adjusted life-years (QALY) for each death was factored into the model. All costs and utilities were discounted at 3% per year, as recommended by the second Panel on Cost-Effectiveness in Health and Medicine [41].

### Cost-effectiveness analysis

A cost-effectiveness analysis was conducted from a healthcare perspective, considering costs as they related directly to health expenditures, and run over a hypothetical one-year time horizon [41]. This differs from a societal perspective, which incorporates a comprehensive assessment of costs and benefits [41]. The primary outcomes evaluated in this study were cost, effectiveness (SBI accurately diagnosed and treated), and cost-effectiveness for each strategy. Strategies were ranked by cost then compared in terms of cost, effectiveness, and incremental cost-effectiveness ratio (ICER). The ICER measures added cost for additional benefit to a population, measured in QALYs, and reflects the value of an intervention. QALYs serve as a composite measure of morbidity and mortality. We assumed a willingness-to-pay of $100,000/QALY gained, a commonly cited threshold for the US healthcare system [41]. A strategy was dominated by another strategy if it was both more costly and less effective. Preferred strategies were those with the highest ICER that did not exceed the willingness-to-pay threshold. Secondary outcomes included hospitalizations, lumbar punctures, and deaths. Findings are expressed as costs, QALYs gained, and cost per QALY gained.

### Sensitivity analyses

We conducted one-way sensitivity analyses to determine if varying any individual parameter across its listed range substantially changed results. Threshold analyses determined the point at which changes to certain input parameters (i.e. disease prevalence, sensitivity or specificity of each diagnostic strategy, or cost of medical management) resulted in a substantial change in the preferred strategy. Structural sensitivity analyses evaluated 1) the impact of empiric ceftriaxone administration in strategies that included testing of urine, blood, and CSF, and 2) the potential impact of contaminated cultures on the cost-effectiveness of each strategy. Probabilistic sensitivity analyses estimated the effect of uncertainties in each parameter. For the probabilistic sensitivity analyses, each variable was assigned a distribution of possible values. Distributions were chosen to reflect the level of certainty, the characteristics of the parameter range, and methodological standards. β distributions were used for probabilities and quality adjustments; γ distributions were used for costs. We then used the model to run 1000 simulations for each strategy. For each individual simulation, the model randomly selected a different value for each variable from its assigned distribution. Findings from the probabilistic sensitivity analysis are reported as cost-effectiveness acceptability frontier curves [42]. These curves show the probability that the cost-effectiveness of optimal strategies will be less than or equal to a given $/QALY amount and reflect uncertainty in the model.

## Results

### Bacteremia

**Fig. 2:**
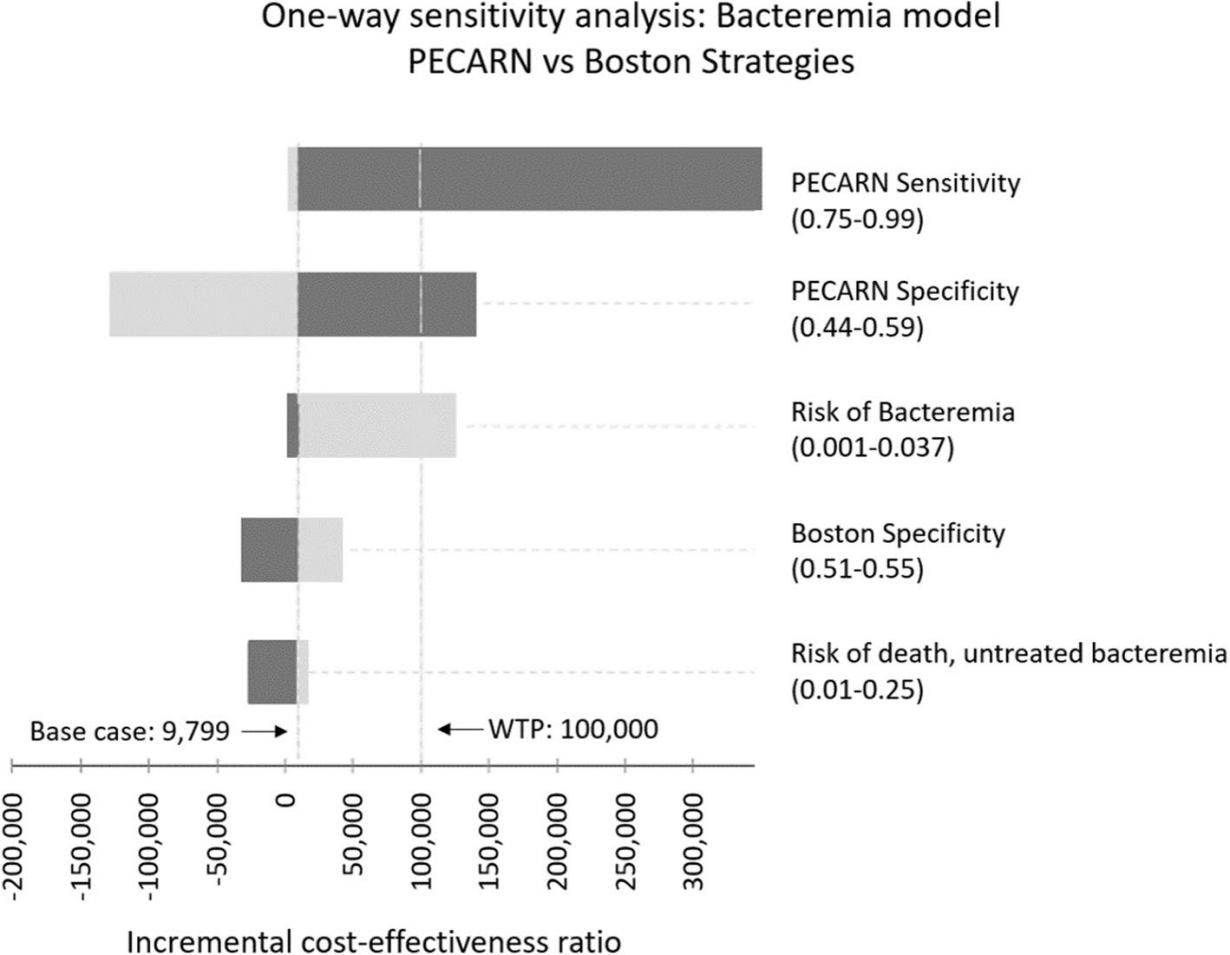
Tornado diagram, one-way sensitivity analysis, bacteremia model. One-way sensitivity analysis of selected variables comparing PECARN and Boston strategies in the bacteremia model. Variables and ranges are listed to the right of the figure. Light gray bars indicate decreasing variable value. Dark gray indicates increasing variable value. The base case, in which the Boston strategy is favored over the PECARN strategy with an incremental cost-effectiveness ratio of $9799/quality-adjusted life-year gained (QALY), is represented in the left vertical dashed line. The willingness-to-pay threshold of $100,000/QALY is represented by the right vertical line. Resulting incremental cost-effectiveness ratio values, in U.S. dollars/QALY, from are represented along the horizontal axis. PECARN, Pediatric emergency care research network; WTP, willingness-to-pay threshold

In the base-case analysis, the PECARN strategy was the least expensive (with a cost of $3671, and a gain of 0.779 QALYs per individual). Compared to the PECARN strategy, the Boston strategy cost $9799/QALY gained. All other strategies were dominated (Table 3). One-way sensitivity analyses demonstrated that the model was sensitive to mortality risk after misdiagnosis, bacteremia prevalence, and the sensitivity and specificity of PECARN, Modified Philadelphia, and Rochester strategies (Table 4, Fig. 2).

**Table 3:** Results of cost-effectiveness analyses, Bacteremia model

	Cost ($)	Incremental Cost ($)	Effectiveness (QALY)	Incremental Effectiveness(QALY)	ICER ($/QALY)
PECARN	$3671	–	0.779	–	–
**Boston**	**$3701**	**$30**	**0.782**	**0.003**	**$9799**
Rochester	$3846	$145	0.778	−0.004	Dominated
Step-by-Step	$3977	$276	0.778	−0.004	Dominated
Clinical suspicion	$4430	$729	0.764	−0.019	Dominated
Aronson	$4527	$826	0.782	−0.001	Dominated
Modified Philadelphia	$4594	$892	0.778	−0.004	Dominated
Philadelphia	$4722	$1021	0.781	−0.001	Dominated

^a^Bold text: Favored strategy at a $100,000 per quality-adjusted life-year threshold

^b^A dominated strategy is more costly and less effective than other strategies

*QALY* quality adjusted life years, *ICER* incremental cost-effectiveness ratio, *PECARN* Pediatric Emergency Care Applied Research Network

**Table 4:** One-way sensitivity analysis results, Bacteremia model

			Preferred Strategy	
Variable	Base-case	Threshold	Below threshold	Above threshold
Risk of bacteremia	0.017	0.005	PECARN	Boston
Risk of death, delayed antibiotics	0.10	0.03	PECARN	Boston
Strategy sensitivity				
PECARN	92%	98%	Boston	PECARN
Strategy specificity				
Boston	53%	52%	PECARN	Boston
PECARN	52%	57%	Boston	PECARN
Rochester	48%	59%	Boston	Rochester
Clinical suspicion	37%	81%	Boston	Clinical suspicion

*PECARN* Pediatric Emergency Care Applied Research Network

In a structural sensitivity analysis, when empiric ceftriaxone administration was removed from the Boston strategy, effectiveness of the Boston strategy decreased to 0.771 QALYs per individual. The PECARN strategy became the preferred strategy and Boston was dominated. Addition of ceftriaxone to the Philadelphia strategy did not change model outcomes. Addition of contaminated cultures with presumptive reevaluation and treatment of affected patients did not change model outcomes. Comparative clinical outcomes in a hypothetical population are shown in Table 5.

**Table 5:** Outcomes by strategy in a population of 1000 febrile infants, of whom 17 have bacteremia

Strategy	Bacteremia Cases, n	Not low risk, Bacteremia, % (n)	Low risk, Bacteremia % (n)	Not low risk, No bacteremia, % (n)	Lumbar Puncture, % (n)	Death from bacteremia, %
Clinical suspicion	17	66 (11)	31 (6)	62 (620)	63 (633)	0.05
PECARN	17	92 (16)	4 (1)	47 (471)	49 (487)	0.01
Modified Philadelphia	17	82 (14)	16 (3)	44 (444)	46 (459)	0.03
Step-by-Step	17	90 (15)	9 (2)	52 (523)	54 (538)	0.02
Boston	17	76 (13)	21 (4)	47 (465)	100 (1000)	< 0.01
Rochester	17	90 (15)	9 (2)	51 (509)	52 (524)	0.02
Aronson	17	97 (17)	3 (0)	63 (631)	65 (648)	< 0.01
Philadelphia	17	97 (17)	3 (0)	66 (657)	100 (1000)	< 0.01

*PECARN* Pediatric Emergency Care Applied Research Network

**Fig. 3:**
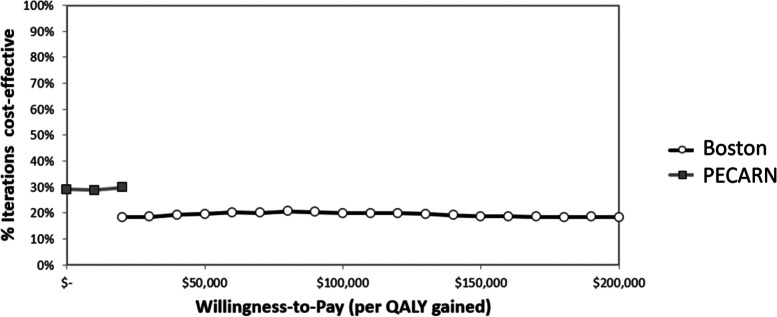
Cost-effectiveness acceptability frontier. Probabilistic sensitivity analysis, bacteremia model. Results are shown as cost-effectiveness acceptability frontier curves, depicting the cumulative probability (y-axis) that favored strategies are favored, compared to other strategies, over a range of willingness-to-pay thresholds (x-axis). The dashed line indicates a willingness-to-pay threshold of $100,000/QALY. PECARN, Pediatric Emergency Care Applied Research Network; QALY, quality-adjusted life-year

**Fig. 4:**
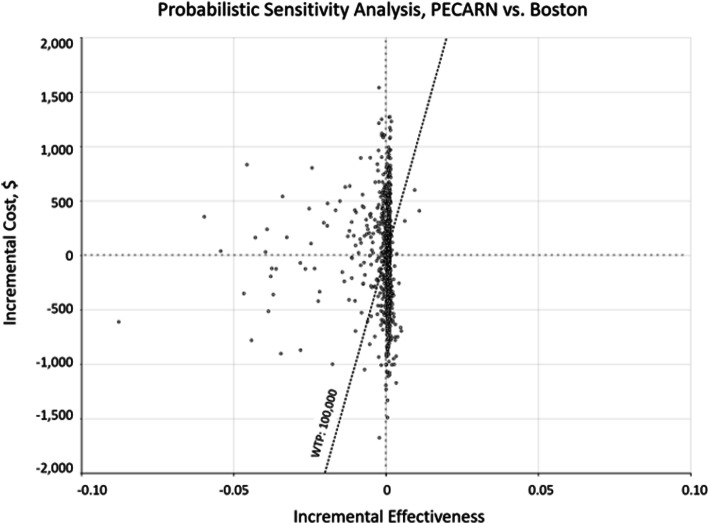
Probabilistic sensitivity analysis, PECARN vs Boston, bacteremia model. Results of the probabilistic sensitivity analysis represented as a scatterplot of the incremental cost (x-axis) and incremental effectiveness (y-axis) of the Boston strategy as compared to the PECARN strategy. The willingness-to-pay threshold of $100,000/quality-adjusted life-year, is indicated by the diagonal dashed line

PECARN, Pediatric Emergency Care Applied Research Network; WTP, willingness-to-pay threshold. 

Probabilistic sensitivity analysis results are summarized as cost-effectiveness acceptability frontier curves, showing the uncertainty associated with the optimal options, calculated using the net monetary benefit framework, over a range of willingness-to-pay (or acceptability) thresholds, as shown in Fig. 3. In this analysis, the Boston strategy was the preferred strategy when the willingness-to-pay was >$10,000/QALY. At a willingness-to-pay of $100,000/QALY, the Boston strategy was the more cost-effective option in 20% of model iterations. A scatter-plot comparing the incremental cost and incremental effectiveness of the Boston strategy to the PECARN strategy demonstrated that the Boston strategy was below the $100,000/QALY threshold in 67% of model iterations (Fig. 4).

### UTI

In the UTI model, the PECARN strategy was the least expensive strategy ($3422, 0.842 QALYs). All other strategies were more expensive and less effective (Table 6).

**Table 6:** Results of cost-effectiveness analyses, Urinary tract infection model

	Cost ($)	Incremental Cost ($)	Effectiveness (QALY)	Incremental Effectiveness (QALY)	ICER ($/QALY)
**PECARN** ^***a***^	**$3422**	**–**	**0.842**	**–**	**–**
Step-by-Step	$3505	$83	0.842	−0.0002	Dominated^*b*^
Boston	$3731	$310	0.838	−0.004	Dominated
Rochester	$3995	$573	0.841	−0.001	Dominated
Clinical suspicion	$4521	$1099	0.837	−0.005	Dominated
Philadelphia	$4774	$1352	0.839	−0.003	Dominated

^a^Bold text: Favored strategy at a $100,000 per QALY

^b^A dominated strategy is more costly and less effective than other strategies

*QALY* quality adjusted life years, *ICER* incremental cost-effectiveness ratio, *PECARN* Pediatric Emergency Care Applied Research Network

One-way sensitivity analyses demonstrated that the Step-by-step strategy would be preferred if it had a specificity for UTI greater than 58% or if specificity of the PECARN strategy was less than 56%. Clinical suspicion was preferred if it had a specificity greater than 67%. Probabilistic sensitivity analyses indicated that at a threshold of $100,000/QALY, the PECARN strategy was preferred in 48% of model iterations.

### Meningitis

For bacterial meningitis, the PECARN strategy was least expensive, and the Boston strategy was preferred with an ICER of $1198/QALY gained. All other strategies were dominated (Table 7). One-way sensitivity analyses demonstrated that this model was sensitive to the specificity of each clinical prediction rule. The PECARN strategy was favored if its sensitivity for identifying bacterial meningitis was greater than 99.8%. Probabilistic sensitivity analysis indicated that at a threshold of $100,000/QALY, the Boston strategy was preferred in 45% of model iterations.

**Table 7:** Results of cost-effectiveness analyses, Bacterial meningitis model

	Cost ($)	Incremental Cost ($)	Effectiveness (QALY)	Incremental Effectiveness (QALY)	ICER ($/QALY)
PECARN	$4290		0.689		
**Boston**	**$4398**	**$108**	**0.779**	**0.090**	**$1198**
Step-by-Step	$4669	$272	0.773	−0.006	Dominated
Aronson	$5015	$617	0.772	−0.007	Dominated
Rochester	$5308	$911	0.772	−0.007	Dominated
Philadelphia	$5466	$1068	0.771	−0.008	Dominated
Clinical suspicion	$5518	$1120	0.734	−0.046	Dominated
Modified Philadelphia	$5824	$1426	0.771	−0.008	Dominated

^a^Bold text: Favored strategy at a $100,000 per quality adjusted life year threshold

^b^A dominated strategy is more costly and less effective than other strategies

*QALY* quality adjusted life years, *ICER* incremental cost-effectiveness ratio, *PECARN* Pediatric Emergency Care Applied Research Network

## Discussion

We performed a cost-effectiveness analysis to compare commonly cited strategies for risk-stratification in the evaluation of febrile infants, finding that Boston and PECARN strategies provided economically reasonable risk stratification strategies compared to other published clinical prediction rules. Models for each type of SBI varied with respect to treatment costs and health risks after misdiagnosis; the PECARN strategy was favored in the UTI model while the Boston strategy was increasingly cost-effective with higher risk infection types.

We found that the Boston strategy was the most cost-effective strategy in both the bacteremia and bacterial meningitis models. Despite lower sensitivity compared to other strategies, the protective effect offered by empiric ceftriaxone and the cost-savings introduced by a higher specificity outweighed the disutility and costs associated with universal lumbar puncture and CSF testing. Alternatively, the PECARN strategy, which does not require CSF testing or empiric antibiotic administration, was an economical option in both models and may provide benefits not measured in this study, depending on individual risk tolerance or preferences.

We found that in the UTI model, most strategies had a sensitivity ≥90% and the risks associated with delayed antibiotics were less substantial than in the other models. Because of this, the benefits of empiric ceftriaxone had a smaller impact, and the model became more sensitive to the costs and disutility associated with admitting patients who were ultimately not diagnosed with UTI.

We found that there were no scenarios in which application of clinical suspicion alone was economically reasonable. In this strategy, infants with fever were assumed to undergo no diagnostic testing if the treating physician assessed their risk to be < 1%. Prior studies investigating variation in infant fever management have noted that a proportion of young febrile infants are discharged from pediatric EDs without additional testing [4]. Our model suggests that the benefits of decreased upfront resource utilization are outweighed by the increased risk and associated costs for the few missed infants with SBI.

To our knowledge, this is the first cost-effectiveness analysis of risk-stratification of febrile infants to evaluate the most recently reported clinical prediction rules for febrile infants. Lieu, et al., demonstrated the benefits of outpatient management of low-risk infants with ceftriaxone using Boston and Philadelphia criteria [29]. In their study, sensitivity analyses indicated that treatment of low-risk infants with ceftriaxone would not be the preferred strategy if an alternative diagnostic strategy had ≥97% sensitivity. In our sensitivity analysis, the PECARN strategy would be preferred over the Boston strategy if its sensitivity were ≥ 98%. Our study builds on prior work by considering the impact of modern disease prevalence and epidemiology, as well as prediction rules with improved diagnostic accuracy.

By simulating the experience of large patient populations, Markov analyses identify strategies that benefit the most individuals most often and lend themselves to broader interpretations. Our study findings may also inform shared decision-making discussions. We found that most strategies have similar effectiveness (Table 3). Strategies that require cerebrospinal fluid testing or result in more frequent hospitalization are more costly but may provide benefits not measured in this study, depending on individual risk tolerance or preferences. One of the strengths of this study is the separate consideration of the three most common serious bacterial infections in infants. While their presentations can be similar and prior studies have analyzed them as a group, the prognosis and the consequences of misdiagnosis for each are substantially different, particularly for bacteremia and bacterial meningitis [8, 30–34]. Recent investigators have also attempted to separate UTI from bacteremia and bacterial meningitis, using the term invasive bacterial infection for the latter [4, 11–14]. In this study, developing disease specific models allowed for a better understanding of how each rule performed across the spectrum of disease, from the low risk and low costs of UTI to the high risk and high costs of bacterial meningitis. The cost utilization of a new consensus guideline provided by the American Academy of Pediatrics is an additional consideration; at the time of this publication, no study has validated this decision rule to determine its performance characteristics [43].

Our findings are subject to limitations. Older prediction rules, such as the Philadelphia, Rochester and Boston criteria, were developed and validated during a period when invasive bacterial infection had a higher incidence [44]. In contrast, some recent rules, such as the Modified Philadelphia, Aronson, and Step-by-Step criteria, may be more reflective of present-day epidemiology but lack external validation. Local practices often do not strictly adhere to published protocols and our model was limited to strategies with published data [4]. We attempted to account for these factors by examining sensitivity and specificity ranges across their calculated 95% confidence intervals in sensitivity analyses. We did not consider the impact of increased outpatient visits associated with a larger proportion of infants categorized as low-risk. Given that our sensitivity analysis did not demonstrate that the model was sensitive to either the large costs of hospitalization or the relatively low costs of CSF testing, it is unlikely that the additional cost of an outpatient visit would change model outcomes.

We adapted utility values from the literature. Infant health state utilities are poorly defined and understudied [45]. It is possible that an older individual’s experience with bacterial infection, lumbar puncture, or hospitalization is different from that of an infant. For this reason, selected utility values were varied over wide ranges. Varying these values did not change favored strategies.

We used a healthcare perspective and, as such, did not evaluate the perspective of families and caregivers, costs of missed or lost employment, or the disutility of caring for an ill child either in the hospital or at home. This, in turn, could bias results toward or against rules associated with higher hospitalization rates. We did not account for inherent risks of hospitalization and medical interventions, including iatrogenic complications and nosocomial infections, and subsequent associated costs. However, these would only increase the costs associated with strategies that require more frequent hospitalization and thereby not change the ultimate findings in our study. Medical costs and willingness-to-pay were based on the U.S. healthcare system and assumptions about the simulated population were drawn primarily from U.S. based data. As such, it is difficult to apply this model to more resource-limited settings. Despite these limitations, this study demonstrates the value associated with application of clincial prediction rules in the emergency setting, and how we can effectively and efficiently evaluate young febrile infants from the perspective of clinicians and health systems.

## Conclusion

In this cost-effectiveness analysis evaluating strategies for the risk-stratification of young febrile infants, we found that the Boston and PECARN clinical prediction rules are economically reasonable strategies compared to alternative strategies when considering outcomes of UTI, bacteremia, and bacterial meningitis. The Boston strategy was more effective and economically reasonable for bacteremia and bacterial meningitis, whereas the PECARN strategy was preferred in UTI. Our findings highlight the benefits of a risk-stratification strategies that avoid potentially unnecessary hospitalizations, either with empiric antibiotic treatment or by maximizing sensitivity and specificity of the initial evaluation.

## Data Availability

All data generated or analyzed during this study are included in this published article [and its supplementary information files].

## Abbreviations

SBI: Serious bacterial infection
PECARN: Pediatric Emergency Care Applied Research Network
UTI: Urinary tract infection
ED: Emergency department
QALY: Quality-adjusted life-years
ICER: Incremental cost-effectiveness ratio
UA: Urinalysis
CBC: Complete blood count
CSF: Cerebrospinal fluid
CRP: C-reactive protein
PCT: Procalcitonin
ANC: Absolute neutrophil count
